# Estimating the Number of Primary vs Incidental COVID-19 Hospitalizations in Santa Clara County

**DOI:** 10.1093/ofid/ofaf078

**Published:** 2025-02-11

**Authors:** Rosamond Smith, Alexis D’Agostino, Pamela Stoddard, Ahmad Kamal, Kate Kelsey, Linlin Li, Wen Lin, Wayne T A Enanoria, Sarah L Rudman, Christopher M Hoover

**Affiliations:** County of Santa Clara Public Health Department, San Jose, California, USA; County of Santa Clara Public Health Department, San Jose, California, USA; County of Santa Clara Public Health Department, San Jose, California, USA; Santa Clara Valley Medical Center, San Jose, California, USA; County of Santa Clara Public Health Department, San Jose, California, USA; County of Santa Clara Public Health Department, San Jose, California, USA; County of Santa Clara Public Health Department, San Jose, California, USA; County of Santa Clara Public Health Department, San Jose, California, USA; County of Santa Clara Public Health Department, San Jose, California, USA; County of Santa Clara Public Health Department, San Jose, California, USA

**Keywords:** COVID-19, epidemiology, hospitalizations, SARS-CoV-2, local public health

## Abstract

**Background:**

The goal of this study was to evaluate whether International Classification of Diseases, 10th Revision (ICD-10), discharge data can be used to accurately differentiate primary coronavirus disease 2019 (COVID-19) hospitalizations, which are specifically due to COVID-19, from incidental COVID-19 hospitalizations for monitoring COVID-19 trends in a large county health department. We sought to explore the use of machine learning algorithms for enhancing surveillance capabilities in a local public health setting.

**Methods:**

Discharge data for 5122 Santa Clara County hospitalizations with a positive severe acute respiratory syndrome coronavirus 2 polymerase chain reaction or antigen test occurring between December 15, 2021, and August 15, 2022, were used to train a series of models for classifying primary COVID-19 hospitalizations using chart review as a gold standard. Area under the receiver operating characteristic curve (AUROC) was used as the evaluation metric.

**Results:**

Each model performed well when trained on the full set of available predictors. AUROC values ranged from 0.808 (random forest) to 0.818 (SuperLearner). After evaluating each model, we implemented a reporting process based on Least Absolute Shrinkage and Selection Operator (LASSO) logistic regression, as the performance was comparable with SuperLearner and it had the advantage of being transparent and familiar to health department staff.

**Conclusions:**

In Santa Clara County, ICD-10 discharge data were successfully used to develop a low-burden method for monitoring primary COVID-19 hospitalization, demonstrating one way that predictive algorithms can help local health jurisdictions meet surveillance needs while minimizing manual effort.

Understanding coronavirus disease 2019 (COVID-19) infection trends, particularly for severe disease, is helpful for communities looking to minimize risk and plan for possible stress on the health care system. However, disease surveillance systems that differentiate between incidental disease and severe disease in hospitalized patients often rely on onerous manual input by health care providers and are not feasible for high-volume diseases. This paper outlines how 1 large local health department addressed this challenge with a simple machine learning (ML) algorithm for classifying primary COVID-19 hospitalizations, which are specifically due to COVID-19 and often indicate severe disease.

As changes in reporting requirements and testing behaviors have decreased the accessibility of testing and case data for COVID-19 surveillance, the Centers for Disease Control and Prevention (CDC) and other public health entities have increasingly relied on COVID-19 hospitalizations as a primary metric for tracking COVID-19 trends and informing planning and prevention measures [1]. As of September 2023, the CDC guidelines dictated that all hospitalized patients with a confirmed positive severe acute respiratory syndrome coronavirus 2 (SARS-CoV-2) test, regardless of the underlying cause of hospitalization, are COVID-19 hospitalizations [2]. This broad definition may overestimate the burden of COVID-19 on hospitals and the community as some patients hospitalized for other reasons (eg, fracture, childbirth) may incidentally test positive for SARS-CoV-2 while they are in the hospital [3]. Tracking primary COVID-19 and incidental COVID-19 hospitalizations separately can assist health officials in assessing the risk of severe disease, issuing appropriate guidance, and informing policy decisions.

Some local and state health jurisdictions, including Los Angeles County, CA, Marin County, CA, and Massachusetts, have defined methods for differentiating primary and incidental COVID-19 hospitalizations [4–6]. Each department uses a different method. Los Angeles County uses a case definition based on the presence of specific International Classification of Diseases, 10th Revision (ICD-10), codes (Supplementary Data), Marin County relies on chart review, and Massachusetts uses a case definition based on dexamethasone treatment [4, 5, 7]. As of August 2023, these departments were reporting between 33% and 48% of all COVID-19 hospitalizations as primary COVID-19 hospitalizations.

These examples are generally representative of the approaches proposed in prior studies including manual chart review, case definitions based on receipt of treatment for COVID-19 (eg, dexamethasone, remdesivir) or symptoms of severe COVID-19 (eg, low SpO2 levels), and electronic health record (EHR) data mining (eg, use of lab test data, ICD-10 codes, procedure codes, clinical notes) [4, 5, 8–10]. Manual chart review in particular creates a substantial reporting burden and may be not feasible for large health jurisdictions outside of an emergency environment due to competing priorities of clinical staff. Furthermore, local and state health jurisdictions may have difficulty accessing all the clinical data points used in prior research from other settings to identify primary COVID-19 hospitalizations; moreover, requesting such data represents a reporting burden for hospitals [11].

The goal of this study was to evaluate whether supervised ML methods can accurately and efficiently distinguish primary from incidental COVID-19 hospitalizations. Using all COVID-19 hospitalizations in Santa Clara County (SCC), an ethnically and socioeconomically diverse county of nearly 2 million in California's San Francisco Bay Area, we identified primary COVID-19 hospitalizations using ICD-10 discharge data, with chart review by a health care provider as a gold standard. Like Los Angeles County, which also used ICD-10 data, we used data that are commonly available to local health jurisdictions. We leveraged information from a period when chart review–based clinical judgments were required for all SARS-CoV-2-positive hospitalizations in SCC. We sought to develop a generalizable workflow that minimizes manual reporting to inform ongoing COVID-19 surveillance and to explore the use of ML for enhancing local public health surveillance capabilities.

## METHODS

### Study Data

Our study relied on 3 data sources: SCC COVID-19 Case Report Form (CRF) submissions for primary COVID-19 hospitalization, data from California's COVID-19 contact tracing database (CalCONNECT), and information from the California Department of Public Health's (CDPH’s) COVID-19 hospital discharge data for SCC, which includes data for all hospitalizations with a positive SARS-CoV-2 test, including both polymerase chain reaction and antigen tests. Hospitals in California are required to report these data to CDPH for all hospitalizations with a positive SARS-CoV-2 test.

Health care providers in SCC were required to submit CRFs to the health department for all COVID-19 cases in certain settings, including hospitals, from April 8, 2020, to September 19, 2022 [12, 13]. Hospital CRFs included a clinical determination of whether each case was a primary COVID-19 hospitalization. Clinical determinations were made at the hospital level by whomever the hospital determined clinically appropriate to make this determination—often infection control nurses but occasionally physicians or other providers—who conducted chart reviews, judged whether each hospitalization was a primary or incidental COVID-19 hospitalization, and completed the CRFs.

Our CRF data had limited information, including first name, last name, date of birth, date of admission, and a unique number used to link cases to CalCONNECT for appending hospital name, street address, and any missing hospital dates of admission. Augmenting the CRFs with data from CalCONNECT also enabled merging to the CDPH data, which contain standard demographic data and ICD-10 discharge codes for all SARS-CoV-2-positive hospitalizations. We combined the CDPH data and the CalCONNECT-augmented CRF data using a series of matching rules looking for combinations of first name, last name, date of birth, date of admission, and street address. CRF records without a match in the CDPH data were excluded. Some excluded CRF records may reflect submissions made without a confirmed positive test result. CRF clinical determinations were used to identify primary COVID-19 hospitalizations in the merged data set. To assess completeness of the final data set, we cross-checked the volume of hospitalizations with COVID-19 patient data submitted directly to SCC by county hospitals. Patients with missing demographic data were excluded from the analysis.

All hospitalizations with a positive SARS-CoV-2 test that occurred in an SCC hospital between December 15, 2021, and August 15, 2022, were included. December 15, 2021, is the week when Omicron became dominant in SCC. We exclude hospitalizations from previous variant waves because clinical characteristics may differ. Previous studies have reported that patients hospitalized during the Omicron period are more likely to have comorbidities and tend to be older than patients hospitalized during the Delta period [14]. The SCC's CRF requirement ended on September 19, 2022, and providers were not required to submit CRFs for hospitalizations before September 19 that had not yet been reported. As CRFs could be delayed for several weeks, we excluded hospitalizations occurring after August 15, 2022.

### Analysis

We trained Least Absolute Shrinkage and Selection Operator (LASSO) logistic regression, random forest, and extreme gradient boosting (XGBoost) models using 10-fold cross-validation to assess whether ICD-10 discharge codes could be used to identify primary (vs incidental) COVID-19 hospitalization. We chose these models because regression models are transparent and likely familiar to department staff, while tree-based models are intuitive and explainable to a variety of audiences. Furthermore, previous reports have shown success applying these methods to study COVID-19 hospitalization and mortality [10, 15–18]. We developed a strawman model for comparison, which used a simple case definition where any hospitalization with the ICD-10 code J1282 (pneumonia due to coronavirus) was classified as primary COVID-19.

We considered 4 sets of predictors. The first set included 4 ICD-10 codes identified through input from several other California county health departments (Supplementary Table A). The second set included 13 ICD-10 codes (Supplementary Table B) identified by a local clinician on our research team. The third set was based on Los Angeles County's COVID-19-associated hospitalization definition (Supplementary Data). The fourth set included all ICD-10 codes associated with at least 50 hospitalizations, the number of ICD-10 codes for each patient, gender, age, length of hospitalization, and number of days between positive test and date of admission. We refer to these feature sets as the subject matter expert (SME) 1, SME 2, Los Angeles County (LAC), and full sets.

For the full set, we also used SuperLearner to train a weighted ensemble ML algorithm that combines the LASSO regression, random forest, and XGBoost models and assigns weights based on how much value each model adds [19–21].

We used AUROC, with 10-fold cross-validation, to select the best model, keeping in mind our goal to improve our COVID-19 surveillance capacity using methods that performed well but were as transparent, explainable, and familiar as possible. Because our data were slightly imbalanced, we also calculated area under the precision-recall curve (AUCPR). For 10-fold cross-validation, we randomly divided the data into 10 groups and held 1 group out as the test set, creating a 90%/10% train–test split. We repeated this hold-out process 10 times, each time using a different group as the test set. We combined the test set results from each round of cross-validation to measure performance. Cross-validation has previously been shown to produce less biased performance estimates compared with a single train/test split approach [22].

### Implementation

Trained models were used to estimate the probability that each hospitalization was primarily due to COVID-19. Considering each hospitalization individually, these algorithms can be assessed using sensitivity, specificity, and accuracy by comparing the prediction for each hospitalization with the gold standard determinations on which each model was trained.

Alternatively, we can use these models to assess population-level impact by summing estimated probabilities for individual hospitalizations over a particular time frame (eg, each week) to determine the expected number of primary COVID-19 hospitalizations in the target population during that time frame. With this approach, we can assess each algorithm using mean squared error (MSE), which compares the expected number of primary COVID-19 hospitalizations with the actual number reported through CRFs during a given time frame. As a public health agency, we were interested in estimating the population-level burden of primary COVID-19 hospitalization, and therefore focused on this approach in the results. We also calculated 95% confidence intervals for our summed weekly estimates using bootstrapping.

### Ongoing Validation

In December 2022, we implemented a report to retrospectively monitor the estimated proportion of primary (vs incidental) COVID-19 hospitalizations each week in SCC hospitals. Because our gold standard data are limited to a specific period, it was important for us to identify a mechanism for continuous performance monitoring. We identified a subset of COVID-19 hospitalizations from the county's health care system with continued clinical judgments, allowing for ongoing validation. From August 16, 2022, through September 30, 2023, we calculated weekly MSE on this subset to track how our estimates were performing and to notify us if they deviated substantially from the clinical judgments.

Analyses were performed in R, version 4.1.3, using the glment, randomForest, xgboost, SuperLearner, caret, and pROC packages [20, 23–28].

The SCC Public Health Department deemed this work public health surveillance; the Revised Common Rule deems “public health surveillance activities” not subject to institutional review board (IRB) oversight under 45 CFR § 46.

## RESULTS

There were 5174 confirmed positive SARS-CoV-2 hospitalizations in the CDPH data with dates of admission between December 15, 2021, and August 15, 2022. After merging in the 2224 CRF records (ie, hospitalizations with primary COVID-19 clinical judgments), we identified 1763 SARS-CoV-2 hospitalizations with an associated primary COVID-19 record in the CRF data, 3411 SARS-CoV-2 hospitalizations with no associated primary COVID-19 record in the CRF data, and 461 primary COVID-19 CRF records with no associated SARS-CoV-2 hospitalization in the CDPH data. The 461 unmatched primary COVID-19 records were excluded. The age distributions of excluded vs included primary COVID-19 records were similar. The median age (interquartile range) of primary COVID-19 patients in the final data set was 71 (55–84) years, compared with 72 (54–83) for excluded primary COVID-19 patients.

**Figure 1.**
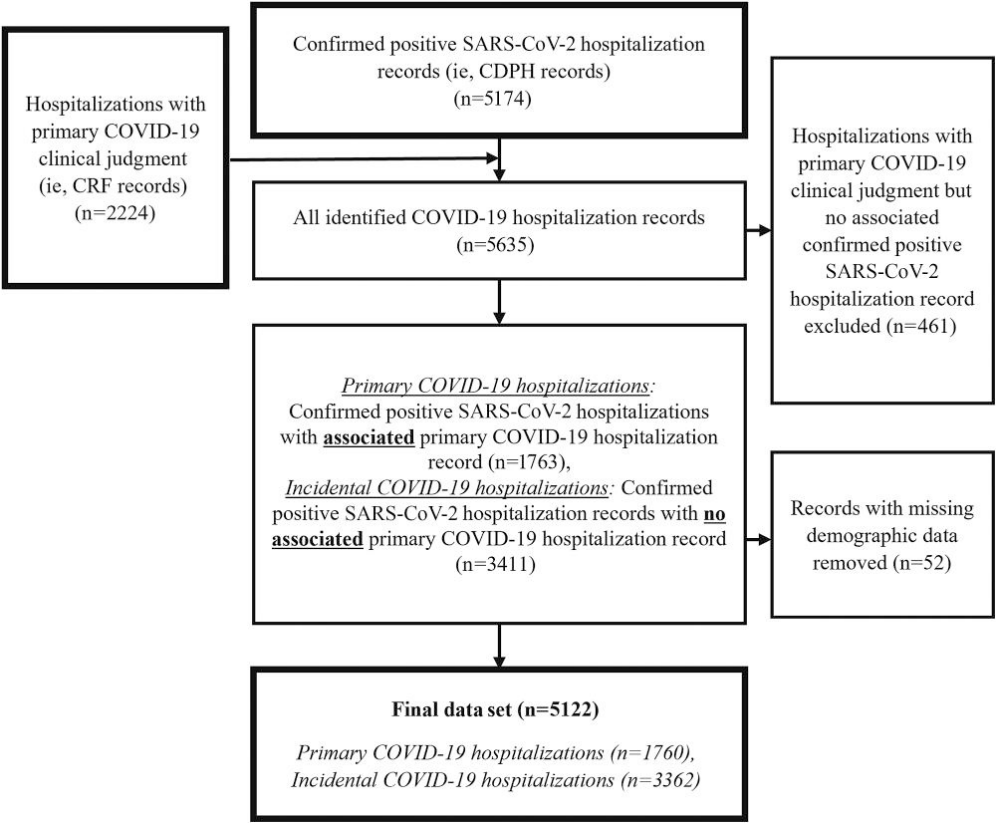
Summary of COVID-19 hospitalization data joining process: Santa Clara County, 12/15/2021–8/15/2022. Abbreviations: CDPH, California Department of Public Health; COVID-19, coronavirus disease 2019; SARS-CoV-2, severe acute respiratory syndrome coronavirus 2.

Fifty-two records were excluded due to missing demographics. The final data set included 5122 SARS-CoV-2-positive hospitalizations, with 1760 (34%) primary COVID-19 hospitalizations. Figure 1 summarizes the data-joining process.

Table 1 provides summary statistics for these 5122 primary and incidental COVID-19 hospitalizations. In our sample, primary COVID-19 patients were older and had longer hospital stays on average. The median age for primary COVID-19 patients was 71, vs 63 for incidental COVID-19 patients. Primary COVID-19 hospitalizations lasted 5 days on average, compared with 4 days for incidental hospitalizations.

**Table 1.** Characteristics of Primary and Incidental COVID-19 Hospitalizations: Santa Clara County, 12/15/2021–08/15/2022

Variable	Primary COVID-19 Hospitalizations	Incidental COVID-19 Hospitalizations
Age		
Median (IQR), y	71 (55–84)	63 (39–78)
No. of ICD-10 discharge codes per hospitalization		
Median (IQR)	16 (11–23)	14 (9–22)
Length of stay		
Median (IQR), d	5 (3–9)	4 (2–7)
Top 10 ICD-10 codes with the largest difference in occurrence rates between primary and incidental COVID-19 hospitalizations		
J1282—Pneumonia due to coronavirus disease 2019	58%	19%
J9601—Acute respiratory failure with hypoxia	37%	12%
U071—COVID-19	95%	74%
A4189—Other specified sepsis	18%	6%
E785—Hyperlipidemia, unspecified	41%	30%
Z8616—Personal history of COVID-19	3%	13%
Z370—Single live birth	1%	10%
Z20822—Contact with and (suspected) exposure to COVID-19	7%	15%
E1122—Type 2 diabetes mellitus with diabetic chronic kidney disease	17%	11%
N179—Acute kidney failure	24%	18%

Abbreviations: COVID-19, coronavirus disease 2019; ICD-10, International Classification of Diseases, 10th Revision; IQR, interquartile range.

Primary COVID-19 hospitalizations had more ICD-10 discharge codes and a higher frequency of COVID-19-related codes including U071 (COVID-19) and J1282 (pneumonia due to coronavirus). Primary COVID-19 hospitalizations had an average of 16 ICD-10 codes, compared with 14 for incidental COVID-19 hospitalizations. Among primary COVID-19 hospitalizations, 95% had U071 and 58% had J1282, compared with 74% and 19% of incidental hospitalizations, respectively.

The full SuperLearner model performed best, with an AUROC of 0.818. The full LASSO, random forest, and XGBoost models also performed well, with AUROCs of 0.810, 0.808, and 0.816, respectively. The SME1, SME2, and LAC models performed lower, with the highest AUROC being 0.727 for the SME2 random forest. Table 2 shows the AUROC, AUCPR, weekly MSE, accuracy, sensitivity, and specificity for the 14 models.

**Table 2.** Classifying Primary and Incidental COVID-19 Hospitalizations, Model Evaluation Metrics: Santa Clara County, 12/15/2021–08/15/2022

Feature Set	Classifier	AUROC	AUCPR	Weekly MSE (Min, Max)	Accuracy, %	Sensitivity, %	Specificity, %
Strawman	All J1282 classified as primary	–	–	107.26 (0.00, 529.00)	73.09	57.67	81.17
LAC	Lasso regression	0.691	0.522	102.50 (0.07, 1225.86)	72.10	61.53	77.63
	Random forest	0.704	0.548	1336.40 (3.40, 1336.40)	72.12	61.93	77.45
	XGBoost	0.691	0.525	101.51 (101.51, 1206.52)	72.22	60.91	78.14
SME 1	Lasso regression	0.711	0.566	95.41 (0.01, 1075.26)	72.16	65.45	75.67
	Random forest	0.720	0.575	101.20 (0.02, 525.23)	72.16	65.45	75.67
	XGBoost	0.712	0.564	94.60 (0.05, 1067.20)	72.16	65.45	75.67
SME 2	Lasso regression	0.715	0.556	101.01 (0.01, 1147.95)	71.20	67.95	72.90
	Random forest	0.727	0.576	101.54 (0.00, 528.45)	71.03	68.81	72.19
	XGBoost	0.718	0.558	96.51 (0.06, 1092.32)	70.68	68.86	71.62
Full	Lasso regression	0.810	0.661	67.97 (0.01, 822.77)	76.59	66.93	81.65
	Random forest	0.808	0.672	85.65 (0.04, 1049.63)	75.11	72.56	76.44
	XGBoost	0.816	0.685	64.27 (0.00, 785.34)	75.77	69.72	78.94
	SuperLearner	0.818	0.685	72.06 (0.01, 907.53)	75.69	71.48	77.90

Abbreviations: AUCPR, area under the precision-recall curve; AUROC, area under the receiver operator characteristic curve; MSE, mean squared error.

We chose to implement the full LASSO regression. Although it did not have the best AUROC, AUCPR, or MSE, we felt that the advantages of a regression model, including transparency, explainability, and familiarity to department staff, outweighed the slight performance increase from SuperLearner.

Looking more closely at the full LASSO regression, the 10 most important ICD-10 codes, listed with their coefficients, were:

1. single live birth (Z370): –1.15;
2. pneumonia due to coronavirus (J1282): 1.02;
3. COVID-19 (U071): 0.86;
4. hypoxemia (R0902): 0.67;
5. acute respiratory failure with hypoxia (J9601): 0.67;
6. acute respiratory distress syndrome (J80): 0.59;
7. other ascites (R188): –0.40;
8. alcohol dependence with withdrawal, unspecified (F10239): –0.37;
9. dependence on supplemental oxygen (Z9981): 0.33;
10. nausea with vomiting, unspecified (R112): –0.31.

Several ICD-10 codes with positive coefficients, including pneumonia due to coronavirus, acute respiratory failure with hypoxia, and acute respiratory distress syndrome, were also in the SME feature sets. However, none of these ICD-10 codes with negative coefficients were in the SME feature sets. The full feature set may have been particularly useful for identifying variables associated with incidental COVID-19 hospitalization.

Because we were interested in using this model as a public health tool to produce population-level estimates, we looked at MSE to further understand the performance of the full LASSO regression, which was 67.97 (range, 0.01–822.77). Weekly MSE compares the expected number of primary COVID-19 hospitalizations each week from the test data with the actual number reported through CRF submissions. The square root of the weekly MSE gives the average number of hospitalizations our estimate is off by. A weekly MSE of 67.97 means that, on average, the expected number of primary COVID-19 hospitalizations is off by 8.24 hospitalizations each week. Our data had an average of 146 weekly SARS-CoV-2-positive hospitalizations and 50 primary COVID-19 hospitalizations.

**Figure 2.**
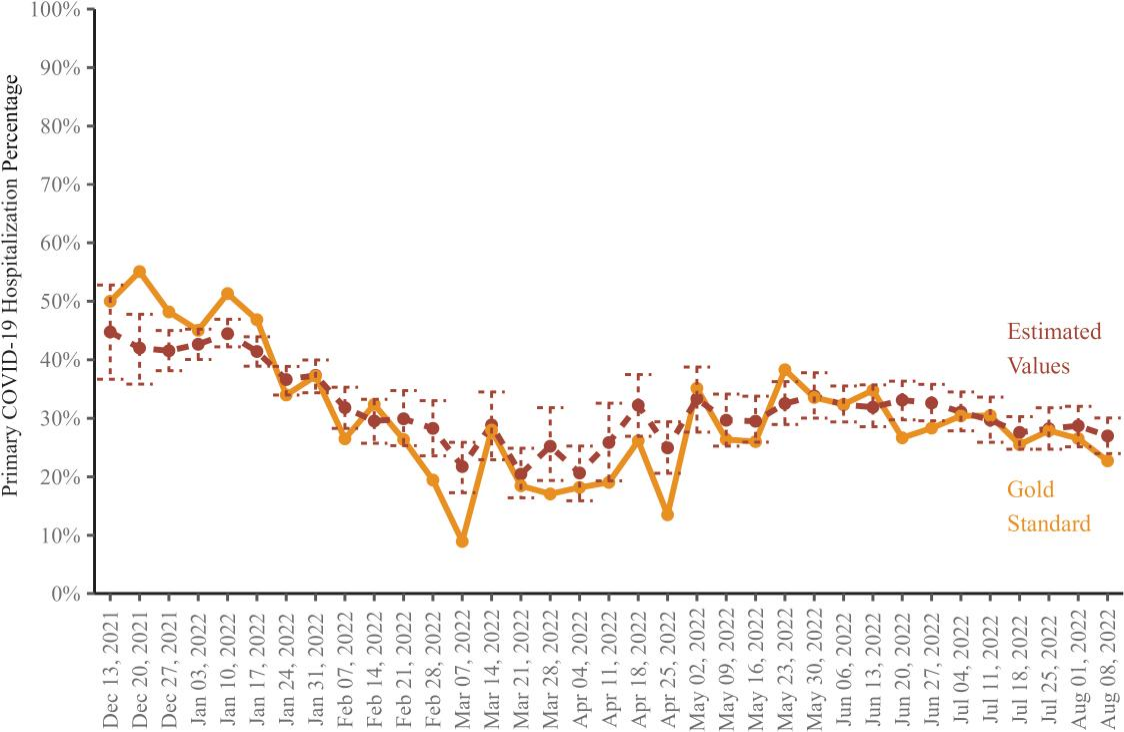
Primary COVID-19 hospitalization percentages by week, gold standard and estimated with 95% confidence intervals: Santa Clara County, 12/15/2021–08/15/2022. Abbreviation: COVID-19, coronavirus disease 2019.

Figure 2 shows the weekly percentage of SARS-CoV-2-positive hospitalizations that were clinically determined to be primary COVID-19 hospitalizations on CRF forms (gold line) compared with LASSO model estimates (red line).

**Figure 3.**
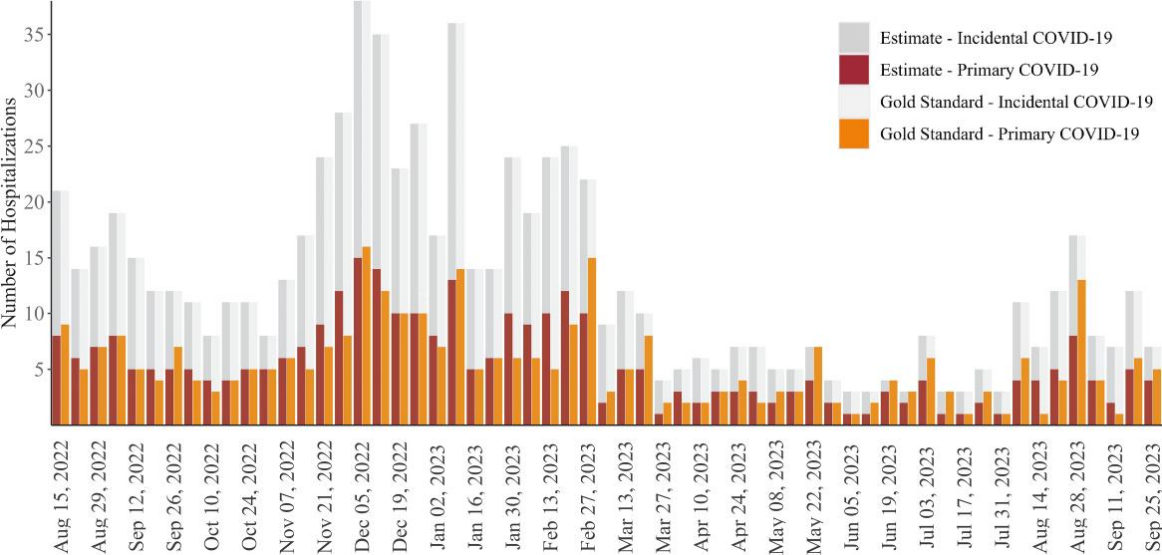
Number of primary and incidental COVID-19 hospitalizations in validation data by week, gold standard and estimated: Santa Clara County, 08/16/2022–09/30/2023. Abbreviation: COVID-19, coronavirus disease 2019.

Using a subset of SARS-CoV-2-positive hospitalizations from the county's health care system, which has continued clinical judgments, we conduct ongoing model validation. Figure 3 shows gold standard and LASSO estimated weekly counts of primary and incidental COVID-19 hospitalization in the validation data, calculated by summing the estimated probabilities for each hospitalization occurring in a given week. Between August 16, 2022, and September 30, 2023, the average weekly MSE (range) was 3.42 (0–25), meaning that our estimates were off by ∼1.85 hospitalizations each week in the validation data, which includes a weekly average of 13 hospitalizations.

## DISCUSSION

COVID-19 will likely be a burden on hospitals for the foreseeable future, and developing locally tailored methods for understanding disease severity as reflected in primary hospitalizations can help in the evaluation of the impact of policy decisions, health behaviors, and other determinants of disease.

Between December 15, 2021, and August 15, 2022, we report that 34% of SARS-CoV-2-positive hospitalizations in our sample were primary COVID-19 hospitalizations. This emphasizes the importance of differentiating COVID-19 hospitalization types. The standard approach of counting all SARS-CoV-2-positive hospitalizations together overestimates the added burden of COVID-19 on our health care system. Though incidental COVID-19 hospitalizations are important to track, as they still suggest increased infection risk and may require extra precautions, monitoring these hospitalizations separately allows for more informed decision-making. This study only considers a binary distinction, when some COVID-19 hospitalizations may require more nuanced classification. Even when COVID-19 is not a primary reason for admission, it could still exacerbate other conditions. Future research should continue to investigate this issue.

While the use of an ensemble algorithm provided a slight performance increase, we show that simpler algorithms, like LASSO regression, performed almost as well. Many health departments may prefer simple models because they are transparent, easy to interpret, and may help secure leadership buy-in for model-based reporting.

This paper outlines one use case for supervised ML in a local health department. This approach allowed us to make the most of our data to estimate an important measure of COVID-19 severity without much added labor. While imperfect, we have successfully used our model to produce estimates of the proportion of primary COVID-19 hospitalizations in SCC.

A similar approach may be useful for other jurisdictions when a gold standard data source is available or can be created. Through this work, we learned several lessons that may help others as they consider applying ML methods. First, having a diverse team providing input on the approach was critical. Our team included decision-makers to assess feasibility and determine appropriate resource allocation, staff who are knowledgeable about affected populations to assess whether our models were exacerbating biases in the initial data, and subject matter, data, and methods experts. Our subject matter experts suggested that we consider all discharge diagnoses rather than only the primary diagnosis, as physicians do not always enter ICD-10 codes in order of clinical importance. Having a team member with deep knowledge of our data allowed us to leverage a third data set (CalCONNECT), improving our data matching and providing a more comprehensive training set. Jurisdictions may consider engaging external partners in cases where department staff do not have the necessary mix of skillsets or expertise. Because our gold standard data were not continuously available, it was important to identify a mechanism for postdeployment monitoring to build trust and flag any emerging performance issues. Considering model estimates in the context of other existing data may also help with building trust and incorporating modeling into routine processes. Together, these factors allow practitioners to include equity considerations in all stages of the modeling lifecycle.

### Limitations

This analysis has several limitations. First, we relied on clinical judgments made at the hospital level as a gold standard for primary COVID-19 hospitalization. We acknowledge that there is subjectivity and variability in these determinations, especially across different clinicians and hospitals. Additionally, SCC's manual reporting process may have been prone to data entry errors, and some CRF submissions had no match in the complete SARS-CoV-2-positive hospitalization data set, meaning that our data may include some classification errors.

We had access to limited data, and some important factors were not explicitly considered, including vaccination and previous infection. There are ICD-10 codes for immunization and history of COVID-19, but these may be undercoded. With more comprehensive data, our model might more accurately capture nuanced relationships between vaccination, previous infection, and primary COVID-19 hospitalization. We also do not account for evolving COVID-19 variants, though we restricted our analysis to the period after the Omicron variant became dominant. Our model is dependent on having no substantial shifts over time that change the underlying relationship between ICD-10 codes and primary COVID-19 hospitalization. These limitations are one reason we conduct ongoing validation, which should alert us to changes in our model's predictive ability. To date, our estimates have continued to track closely with clinical judgments in our validation data.

Another limitation is our use of ICD-10 data. While commonly used for surveillance due to availability and low cost, ICD-10 data vary widely in their accuracy and completeness [29, 30]. Undercoding of some ICD-10 codes and overcoding of others are common. Coding patterns can differ by hospital due to factors including coding practices and EHR builds. Other data sources may be more reliable for this problem, but because we were working to address a challenge quickly and efficiently, we chose to use the data that we had.

Finally, our model relies on discharge data processed by CDPH and then shared with us. This means we can only report retrospectively, and if reporting processes change and we lose access to some data elements, we may no longer be able to use this model. In the future, it may be possible to access the required data (eg, SARS-CoV-2 test results, ICD-10 data) through other sources such as EHR data.

## PUBLIC HEALTH IMPLICATIONS

Local health jurisdictions need methods to minimize the surveillance burden on providers and staff while continuing to monitor COVID-19 in their communities. This study demonstrates one method for estimating primary vs incidental COVID-19 hospitalization and provides an example of how predictive algorithms can help local health jurisdictions meet surveillance needs while minimizing manual effort. While the specific algorithm we discuss was trained on SCC data and may not be generalizable to other jurisdictions, we describe a model-based approach that may be useful for addressing a variety of challenges in local public health settings.

## Supplementary Material

ofaf078_Supplementary_Data

## Supplementary Data

Supplementary materials are available at *Open Forum Infectious Diseases* online. Consisting of data provided by the authors to benefit the reader, the posted materials are not copyedited and are the sole responsibility of the authors, so questions or comments should be addressed to the corresponding author.
