# Supplementary material for: Estimating the Number of Primary vs Incidental COVID-19 Hospitalizations in Santa Clara County
Source: Open Forum Infect Dis. 2025 Feb 11;12(3):ofaf078. doi: 10.1093/ofid/ofaf078 (PMC11884784; doi:10.1093/ofid/ofaf078)
Supplement: ofaf078_Supplementary_Data [file ofaf078_supplementary_data.docx]

## Supplemental Materials

**Table A. Subject Matter Expert 1 Feature Set ICD-10 codes**

| **ICD-10 Code** | **Description** |
| --- | --- |
| J9601 | Acute respiratory failure with hypoxia |
| J1282 | Pneumonia due to coronavirus disease 2019 |
| A4189 | Other specified sepsis |
| A419 | Sepsis, unspecified organism |

**Table B. Subject Matter Expert 2 Feature Set ICD-10 codes**

| **ICD-10 Code** | **Description** |
| --- | --- |
| A4189 | Other specified sepsis |
| A419 | Sepsis, unspecified organism |
| J12 | Viral pneumonia, not elsewhere classified |
| J15 | Bacterial pneumonia, not elsewhere classified |
| J16 | Pneumonia due to other infectious organisms, not elsewhere classified |
| J17 | Pneumonia in diseases classified elsewhere |
| J18 | Pneumonia, unspecified organism |
| J20 | Acute bronchitis |
| J22 | Unspecified acute lower respiratory infection |
| J80 | Acute respiratory distress syndrome |
| J96 | Respiratory failure, not elsewhere classified |
| R0501 | Acute cough |
| R060 | Dyspnea |

**Los Angeles County ICD-10 Method**

Los Angeles County also uses ICD-10 discharge codes to identify primary COVID-19 hospitalization, which they refer to as COVID-19 associated illness. The Los Angeles County case definition is determined based on the presence of a positive SARS-CoV-2 test and a pneumonia, acute respiratory distress syndrome, or acute cardiopulmonary ICD-10 discharge diagnosis.^4^

**Model Training and Evaluation Details**

For our LASSO regression, we determined the value of the tuning parameter, lambda, using a grid search and maximizing the cross-validated area under the receiver operator characteristic curve (AUROC). For the random forest, we set the number of trees equal to 500 and determined the number of randomly selected predictor variables at each node with a grid search that maximized the cross-validated AUROC. For the XGBoost, we tuned the number of trees, depth of trees, and learning rate using a grid search that maximized the cross-validated AUROC.

For model evaluation, we calculated the optimal cutoff threshold in the predicted probability using Youden’s index, a measure which balances sensitivity and specificity, and classified any estimated probability greater than that threshold as a primary COVID-19 hospitalization.
